# Brain Lymphatic Dysfunction in Subarachnoid Hemorrhage: Pathophysiology and Clinical Implications

**DOI:** 10.3390/biom16040616

**Published:** 2026-04-21

**Authors:** Shuangyi Guo, John H. Zhang, Warren Boling, Lei Huang

**Affiliations:** 1 Department of Neurosurgery, Taihe Hospital, Hubei University of Medicine, Shiyan 442000, China; guo.shuangyi@163.com; 2 Department of Physiology and Pharmacology, Basic Sciences, School of Medicine, Loma Linda University, Loma Linda, CA 92354, USA; 3 Department of Neurosurgery, School of Medicine, Loma Linda University, Loma Linda, CA 92354, USA

**Keywords:** subarachnoid hemorrhage, glymphatic system, meningeal lymphatic vessels, cerebrospinal fluid clearance, early brain injury, delayed cerebral ischemia, venous congestion, aquaporin-4, neuroinflammation, hydrocephalus, cognitive impairment

## Abstract

Aneurysmal subarachnoid hemorrhage (SAH) remains a devastating cerebrovascular disorder with high morbidity and mortality, despite advances in aneurysm securing and neurocritical care. Clinical outcomes are determined by early brain injury (EBI), delayed cerebral ischemia (DCI), hydrocephalus, and long-term cognitive impairment, extending beyond the traditional focus on large-vessel vasospasm alone. Emerging evidence identifies the dysfunction of the glymphatic system and meningeal lymphatic pathway, the brain’s primary clearance pathways, as a central and unifying mechanism linking acute hemorrhagic injury to delayed and chronic neurological sequelae. Following SAH, acute intracranial pressure elevation, subarachnoid blood clot burden, loss of arterial pulsatility, venous congestion, astrocytic aquaporin-4 perivascular depolarization, and neuroinflammation converge to suppress cerebrospinal fluid–interstitial fluid exchange and outflow in glymphatic system and subsequent meningeal lymphatic drainage. Persistent clearance failure promotes the retention of blood breakdown products, inflammatory mediators, and metabolic waste, amplifying microvascular dysfunction, cortical spreading depolarizations, blood–brain barrier disruption, and secondary ischemic injury. Importantly, accumulating data highlight venous pathology and meningeal lymphatic impairment as critical, yet underappreciated, contributors to delayed injury and post-SAH hydrocephalus. In this review, we synthesize the current knowledge of the physiological organization of glymphatic and meningeal lymphatic systems, delineate the mechanistic and molecular drivers of their dysfunction after SAH, and discuss clinical implications for EBI, DCI, hydrocephalus, and long-term cognitive outcomes. We further outline future directions, including translational imaging, biomarker development, and therapeutic strategies targeting clearance pathways, to advance disease-modifying approaches in SAH.

## 1. Introduction

Subarachnoid hemorrhage (SAH) is a devastating neurovascular disease associated with high morbidity and mortality. The most common etiology results from an intracranial aneurysm rupture, leading to the abrupt extravasation of arterial blood into the subarachnoid space. The pathophysiology of SAH is multiphasic and multifactorial, evolving through early brain injury (EBI) and delayed secondary injury [1]. These processes involve complex and interrelated vascular, inflammatory, and cerebrospinal fluid (CSF) disturbances [1]. Advances in microsurgical and endovascular techniques have significantly improved early aneurysm securing and initial survival after rupture. However, effective pharmacological and non-surgical strategies to attenuate EBI, prevent delayed cerebral ischemia (DCI), and mitigate long-term neurological sequelae remain limited and continue to be a major focus of basic science and translational research in SAH.

Over the past decade, landmark discoveries of the glymphatic system and meningeal lymphatic vessels (mLVs) in rodents have fundamentally reshaped the understanding of CSF circulation and brain waste clearance pathways as well as brain immunity. The glymphatic system serves as a glia-dependent perivascular network that facilitates CSF–interstitial fluid (ISF) exchange and metabolic waste clearance, functioning predominantly during sleep [2,3,4]. The characterization of functional mLVs demonstrates that they form a critical dural network that bridges the central nervous system (CNS) with peripheral immunity. They drain CSF, metabolic waste, and macromolecules into deep cervical lymph nodes (dcLNs), facilitating immune cell trafficking between the CNS and periphery [5,6,7,8]. These interconnected systems have been shown to play essential roles in fluid homeostasis, metabolic waste clearance, immune surveillance in brain physiologically and pathologically [7,8,9].

Because of its direct anatomical proximity to the brain lymphatic network, SAH is likely one of the stroke subtypes with the greatest impact on the brain’s lymphatic function. Emerging experimental evidence implicates the glymphatic system and mLVs’ dysfunction as critical mediators of post-SAH pathological cascades, including EBI, cerebral edema, neuroinflammation, DCI, and hydrocephalus [10,11,12,13,14,15,16,17,18,19,20,21,22]. Accordingly, restoring glymphatic–meningeal lymphatic function may represent a promising supplemental therapeutic strategy for the management of SAH.

A PubMed search was conducted for studies published between 1 January 2010, and 30 January 2026. The keywords included “glymphatic system”, “meningeal lymphatic vessels”, “cerebrospinal fluid clearance”, “subarachnoid hemorrhage”, “early brain injury” “delayed cerebral ischemia”, “hydrocephalus”, “cognitive impairment”, “venous congestion”, “blood–brain barrier”, “neuroinflammation”, and “AQP4”, used in various combinations. Studies were selected based on their relevance to the glymphatic system and meningeal lymphatics, and the findings were related to SAH and its complications, including both animal and human research. This review summarizes the physiological organization and regulatory mechanisms of the glymphatic and meningeal lymphatic systems, highlights experimental evidence of their dysfunction following SAH, and discusses the emerging translational and clinical implications of targeting the brain lymphatic system for the management of patients with SAH.

## 2. Anatomy and Function of the Brain Lymphatic System

### 2.1. Glymphatic System

The glymphatic system is a brain-wide perivascular network responsible for the exchange of CSF and ISF, enabling the clearance of metabolic waste, excess fluid, and neurotoxic byproducts from the CNS. It was first conceptualized in mice by Nedergaard’s group in 2012. The researchers used two-photon imaging of small fluorescent tracers to monitor the real-time flow of subarachnoid CSF into and through the brain parenchyma in mice. The term “glymphatic system” was introduced to emphasize its dependence on glial cells and its functional resemblance to the peripheral lymphatic system [2]. Subsequent studies have further characterized its function, regulation, and implications in diseases such as Alzheimer’s disease, using imaging in rodents and analyses of post-mortem human samples [2,3,23,24,25]. Neuroimaging biomarkers also support the existence of the glymphatic system in humans [26,27,28,29,30,31,32,33,34,35].

The glymphatic system consists of three sequential compartments: (1) the periarterial influx pathway, (2) parenchymal CSF–ISF exchange spaces, and (3) perivenous efflux routes. CSF from the subarachnoid space enters the brain along periarterial spaces surrounding penetrating arteries, driven by arterial pulsatility and pressure gradients [2,3,4]. These periarterial spaces are bounded externally by astrocytic endfeet and internally by the vascular basement membrane, forming low-resistance conduits for CSF movement. Once within the brain parenchyma, CSF exchanges with ISF across astrocytic endfeet, a process that is critically dependent on perivascularly located aquaporin-4 (AQP4) water channels [2]. Proper localization of AQP4 to perivascular membranes is essential for efficient CSF–ISF exchange. Genetic deletion or the pathological depolarization of AQP4 markedly reduces glymphatic transport efficiency, resulting in impaired clearance of solutes such as amyloid-β, lactate, and inflammatory mediators [2,9,25,36,37]. Following CSF-ISF exchange, interstitial solutes and fluid are subsequently cleared along perivenous spaces, and ultimately efflux into the subarachnoid space, from which they are eliminated through three major routes [37,38]: (1) drainage via mLVs along the dura mater to the cervical lymph nodes; (2) outflow along cranial and spinal nerve sheaths; and (3) direct absorption into the venous circulation through arachnoid granulations. The presence and relative contribution of a direct venous drainage pathway in humans remain controversial, as arachnoid granulations are absent in childhood and inconsistently present in adults [39,40].

Perivascular spaces (PVSs), including periarterial and perivenous compartments, have historically been referred to as Virchow–Robin spaces (VRSs). These are anatomical and CSF-filled channels that surround penetrating cerebral vessels [41]. Although the terms are sometimes used interchangeably, an important distinction exists. VRS primarily denotes the anatomical perivascular space as visualized on histology or neuroimaging, whereas PVS in the context of the glymphatic system emphasizes a functional pathway through which CSF enters along periarterial spaces, exchanges with interstitial fluid, and exits along perivenous routes to facilitate metabolic waste clearance. The perivascular space transitions from a convective, open conduit into a diffusive, restricted, basement membrane-mediated pathway to enable waste clearance at the capillary level. Electron microscopy of the mouse brain identified true capillaries in which astrocyte endfeet directly contact the basement membrane, without the pial sheath seen in conventional PVS [41]. At the capillary level, CSF enters astrocytic endfeet via polarized AQP4, mixes with ISF to generate glymphatic flow, and exits through perivenous spaces toward mLVs for metabolic waste clearance; alternatively, waste drains along basement membranes via intramural periarterial drainage (IPAD) [9,41]. Perivascular spaces form a network of spaces that facilitate CSF transportation and metabolic waste drainage, and are a key structural component of the glymphatic system [2,42]. In addition, the glymphatic system and the blood–brain barrier (BBB) are structurally interconnected through the neurovascular unit (NVU), a complex brain unit composed of neurons, astrocytes, microglia, pericytes, and endothelial cells [43]. While astrocytic endfeet form the outer boundary of the perivascular space, acting as a “glia limitans” that surrounds the entire cerebrovasculature (arterioles, capillaries, and venules), pericytes are located on the inner boundary of the perivascular space, embedded directly within the basement membrane that covers the abluminal surface of endothelial cells. Both astrocytes and pericytes act as “gatekeepers” for blood flow and are crucial for the development and structural integrity of the BBB. Pericytes also promote AQP4 polarization in astrocyte endfeet, implicating their roles in maintaining the correct location of the astrocyte water channels [44]. The glymphatic system functions as a longitudinal, convective clearance pathway for cerebrospinal and interstitial fluids, whereas the BBB serves as a lateral, selective permeability barrier regulating molecular exchange between the circulation and brain parenchyma [43].

Glymphatic transport is an active, regulated process rather than passive diffusion and depends on multiple physiological driving forces to propel CSF along periarterial spaces, with respiratory and vasomotor oscillations further contributing to fluid movement [3,4,45]. Glymphatic activity is enhanced during sleep, coinciding with reduced noradrenergic tone and expansion of the interstitial space [46]. Body posture and intracranial pressure gradients also modulate CSF distribution and clearance efficiency [47,48].

### 2.2. Meningeal Lymphatic Vessels (mLVs)

mLVs were definitively identified in mice in 2015. In mice lacking dural lymphatic vessels, macromolecule clearance from the brain and transport to dcLNs were impaired, while ISF pressure and brain water content were unchanged, indicating direct CSF drainage to dcLNs via dural lymphatics [5]. Using lymphatic endothelial markers and electron microscopy, the immune cells were observed to reside in mLVs [6]. Subsequent studies confirmed the existence of mLVs in humans, using contrast-enhanced MRI in vivo and immunohistochemical analysis of human dura mater [26,49]. mLVs closely resemble initial lymphatic vessels, functioning as conduits for CSF and immune cell entry or drainage into dcLNs [50]. In both mice and humans, mLVs align with dural venous sinuses, but not nasal CSF outflow, and extend anteriorly around the cavernous sinus, exiting via emissary foramina [51]. Animal studies suggest that approximately half of CSF drains through mLVs, while the remaining fraction is cleared along spinal drainage pathways to the mediastinal, iliac, and sacral lymph nodes [8].

mLVs share canonical molecular markers with peripheral lymphatics, including endothelial hyaluronan receptor 1 protein (LYVE-1), podoplanin, vascular endothelial growth factor receptor 3 (VEGFR-3), prospero Homeobox 1(PROX1), and chemokine (C-C motif) ligand 21 (CCL21), but exhibit region-specific differences in size, valve distribution, and transport capacity [7,8]. Unlike peripheral lymphatics, which are largely established during embryogenesis, mLVs develop predominantly after birth, coinciding with increases in intracranial pressure (ICP). The rapid expansion of cerebral blood volume and CSF leads to a marked rise in ICP during human infancy, or the first few postnatal weeks in mice [52]. Meanwhile, mLVs progressively expand along the venous sinuses in a basal-to-dorsal pattern, extending from the skull base toward the confluence of sinuses [39]. Immunohistochemistry using the lymphatic endothelial cell markers in human dura tissue across various regions demonstrated that mLVs are most commonly observed as remote from blood vessels, although they are also found alongside them, with the greatest abundance observed at the skull’s base [49].

As for the location, mLVs are distributed along both convexity (dosal) and skull-base (basal) dura mater in mice and humans [51], each exhibiting unique characteristics [53,54]. Dorsal mLVs, located near the superior sagittal and transverse sinuses, are embedded within the dura and relatively distant from the subarachnoid space. In contrast, basal mLVs, situated along skull-base venous sinuses and major arteries, lie closer to the subarachnoid space, possess lymphatic valves, and serve as major sites for CSF macromolecule clearance. There is a lack of reported data on mLVs in the falx cerebri and tentorium cerebelli. Despite these regional differences, dorsal and basal mLV networks are interconnected and ultimately exit the cranial compartment at the skull base [8,53,54,55].

mLVs support brain homeostasis by mediating waste clearance and CNS immune surveillance [7,50,55,56,57]. By trafficking antigen-presenting cells, T cells, and soluble antigens to cervical lymph nodes, mLVs connect the CNS with the peripheral immune system while preserving immune privilege [56].

## 3. Brain Lymphatic System Dysfunction in SAH

Glymphatic [10,12,13,15,16,17,21,58,59,60,61] and mLVs’ dysfunctions [13,18,19,20,22,62] have been reported in animal models of SAH and have been recently visualized in a small cohort of human patients using a contrast-enhanced MRI technique [29]. SAH pathogenesis and the ensuing brain lymphatic dysfunction constitute a vicious feedback loop that drives pathological progression and leads to deleterious outcomes.

### 3.1. Mechanisms Underlying Brain Lymphatic Dysfunction After SAH

**Figure 1:**
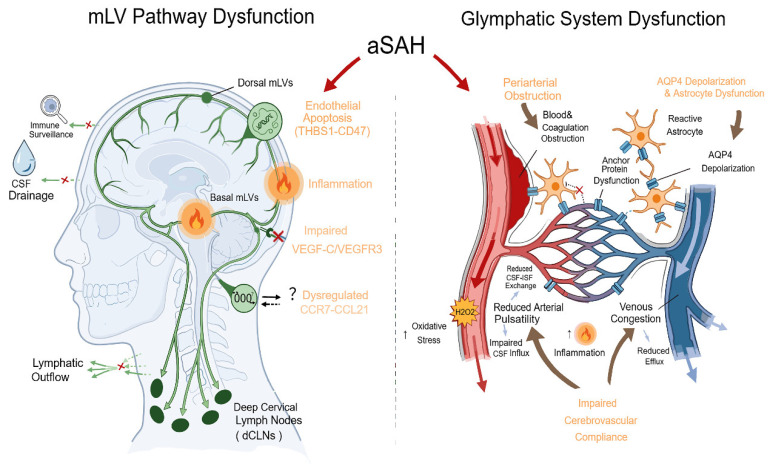
Potential mechanisms affecting the meningeal lymphatic (mLV) pathway and the glymphatic system following aneurysmal subarachnoid hemorrhage (aSAH). “x” indicates blockage or function impairment; “?” indicates a possible signaling in Th17 cells drainage.

The physical obstruction of drainage pathways by blood clots, together with blood toxicity, BBB disruption, neuroinflammation, impaired cerebrovascular compliance, and dysregulated molecular signaling, contribute to dysfunctions in the glymphatic system and mLVs’ pathway after SAH (Figure 1).

#### 3.1.1. Acute Mechanical Obstruction and Blood Toxicity

##### Impact on Glymphatic System

Immediately after intracranial aneurysm rupture, extravasated blood rapidly fills the subarachnoid space, particularly the basal cisterns [63] and extends into periarterial spaces [19], physically obstructing CSF conduits and acutely suppressing periarterial CSF influx into the brain parenchyma. This obstruction coincides with a sudden ICP surge, which may further compress perivascular spaces and collapse glymphatic conduits [64].

In animal models, fluorescently labeled erythrocytes were detected in the subarachnoid space, ventricles, and perivascular spaces around penetrating arterioles within 10 min after prechiasmatic cistern injection. By 2–5 days after SAH, fluorescence was largely confined to leptomeningeal and perivascular macrophages. Erythrocytes also extended into deep perivascular spaces, indicating that perivascular blood can affect both superficial and deeper cortical and subcortical regions after SAH [19]. Other groups have found evidence of erythrocytes and/or blood products in perivascular spaces after SAH in animal models and human patients [11,12]. Beyond red blood cell obstruction that is largely confined to the basal subarachnoid cisterns and to periarterial spaces, hemoglobin degradation products (e.g., hemin) may further diffuse or be transported into the deeper glymphatic pathway. After intracisternal injection in mice, a fluorescent marker with a molecular weight similar to hemoglobin preferentially remained in the peri-arteriolar space, whereas a smaller, heme-sized tracer penetrated into the capillary membrane [65]. In addition, the activation of the coagulation cascade with fibrin deposition in the subarachnoid and perivascular spaces may obstruct CSF-ISF exchange, triggering neuroinflammatory responses and likely contribute to glymphatic dysfunction after SAH. Fibrin deposits were observed on the brain surface including in areas without visible blood [66], and intracisternal fluorescein isothiocyanate (FITC) fluorescent-Dextran accumulation in the cerebellar perivascular spaces was associated with fibrin/fibrinogen presence in perivascular spaces [10] in SAH mice. In a non-human primate SAH model, blood accumulated beneath the ventral arachnoid, with fibrinogen presenting in perivascular spaces, suggesting the clot-mediated occlusion of glymphatic CSF pathways [12].

Consistent with SAH animal models, the intact erythrocytes are found in the perivascular space of post-mortem SAH patients, supporting blood entry into the perivascular space in humans [11]. In CSF and blood samples collected from SAH patients, elevated levels of inflammatory cytokines and extrinsic coagulation pathway factors were observed [67,68,69].

##### Impact on mLVs

After SAH, mLVs are directly exposed to extravasated blood and toxic hemoglobin breakdown products in the subarachnoid space, and actively participate in erythrocyte clearance in animal models [14,21]. Thrombospondin-1 (THBS1) is released from immune cells and activated platelets. The THBS1-CD47 L-R pair promoted mLECs apoptosis via STAT3/Bcl-2 signaling. Genetic or pharmacological inhibition of THBS1–CD47 signaling preserves mLV integrity and improves lymphatic drainage and neurological outcomes, whereas THBS1 overexpression exacerbates mLV injury in SAH mice, implicating THBS1–CD47 signaling as a key mechanistic driver of mLV damage after SAH [20].

#### 3.1.2. BBB Disruption and Neuroinflammation

BBB dysfunction occurs within hours after SAH and is associated with brain edema, microthrombosis, inflammation, and altered cerebral metabolism [70]. In patients with aSAH, elevated serum tight junction protein Claudin-5 levels, the most abundant tight junction protein of the BBB [71], have been shown to correlate with hemorrhage severity [72]. Given that BBB transport and glymphatic clearance work as complementary mechanisms, BBB disruption and glymphatic dysfunction interact [43,73]. BBB damage permits the infiltration of peripheral immune cells and inflammatory mediators into the brain parenchyma, establishing a self-amplifying cycle between BBB disruption and neuroinflammation. Those pathological processes promote reactive astrogliosis and AQP4 depolarization [74], which in turn further exacerbate BBB dysfunction and inflammation after SAH [74].

Perivascular polarization of astrocytic AQP4 is essential for efficient glymphatic function [2,16,17]. Animal experiments have shown that SAH alters astrocytic Ca^2+^ signaling and induces asymmetric hypertrophy of astrocytic endfeet [75,76]. Astrocytes are activated toward a pro-inflammatory A1 phenotype [77], accompanied by endfeet remodeling [13], the loss of perivascular AQP4 polarization [74], distorted endfeet around the capillary, and increased astrocyte apoptosis [65]. In a SAH model of intracisternal blood injection, reduced expression of a component required for assembling the dystrophin-associated complex (DAC), which anchors AQP4 at endfeet, may impair DAC formation and contribute to perivascular AQP4 depolarization [58].

#### 3.1.3. Neurovascular Dysfunction

Cerebral arterial pulsatility is a key driver of paravascular CSF influx into and through the brain parenchyma [3]. After SAH, there are elevations in ICP, occurring acutely (within 24 h), subacutely (up to 7–10 days), or in a delayed phase [59,78]. Because the cranial cavity is a semi-closed compartment, increases in ICP disproportionately impair cerebrovascular compliance [58]. In a balloon model of elevated ICP with cisterna magna tracer injection, glymphatic flow, cerebrovascular pulsatility, and meningeal/deep cervical lymphatic drainage were impaired, with compensatory spinal outflow to sacral lymph nodes proportional to ICP severity [79].

While large arteries drive fluid into the brain, the capillary–perivascular–astrocyte unit is the primary site of waste clearance, making it essential for brain homeostasis [2,9]. At the capillary level, overexpression of matrix metalloproteinase-9 and reduced collagen type IV in the vascular basement membrane compromise membrane integrity and impair vascular function in SAH rats [65].

The perivenular pathway is the primary efflux route of the glymphatic system. Increasing evidence highlights venous-specific changes after SAH, underscoring the importance of venous drainage in disease progression [80,81]. Venous dominance is associated with aneurysm laterality, and venous hypertension may contribute to aneurysm rupture in patients [82,83]. In rabbit models of SAH, vasospasm occurs not only in the basilar artery but also in the deep cerebral veins [57]. These results are consistent with early clinical observations showing elevated basal vein flow velocity correlated with preserved neurological function, whereas reduced flow velocity predicted persistent deficits in SAH patients [84,85]. A recent clinical study further demonstrated that impaired sphenoparietal venous drainage was associated with an increased risk of vasospasm in patients with aSAH [86]. Therefore, venous flow stagnation, elevated venous pressure, and reduced venous compliance may raise interstitial hydrostatic pressure, collapsing perivenous spaces and impairing glymphatic efflux.

Concurrently, venous hypertension may affect the remodeling of mLVs. In mice, bilateral jugular vein ligation (JVL) reproduced intracranial hypertension, mLV regression, and impaired CSF clearance. mLVs remodeling induced by JVL was mediated by vascular endothelial growth factor (VEGF)-C signaling between dural mesenchymal and lymphatic endothelial cells [87]. MRI studies in patients with idiopathic intracranial hypertension have linked dural venous stenosis to impaired lymphatic drainage and brain fluid accumulation [87].

### 3.2. Contribution to SAH Pathogenesis

**Figure 2:**
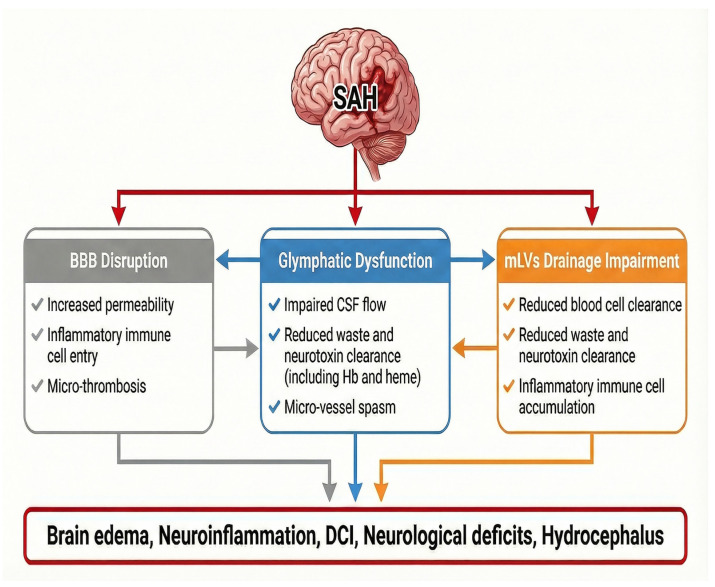
Blood–brain barrier (BBB), glymphatic system, and meningeal lymphatic (mLV) drainage in the pathophysiology of SAH.

Recognition of glymphatic and mLVs dysfunction as integral components of SAH pathophysiology has important implications for biomarker development, clinical risk stratification, and therapeutic advancement. Impaired brain clearance pathways, along with BBB disruption, provide a unifying framework linking EBI to DCI, hydrocephalus, and long-term cognitive impairment (Figure 2).

#### 3.2.1. Time Course

A few studies have characterized the temporal profiles of the glymphatic system and mLV dysfunction after SAH in animal models [20,21,58] and human patients [29].

##### Glymphatic System

In a mouse SAH model of intracisternal blood injection, CSF tracer movement along perivascular spaces into the brain parenchyma was assessed histologically using an intracisternal fluorescent tracer [58]. Glymphatic influx acutely collapsed, peaking at 6 h, and remained impaired through days 1 to 3, with recovery by day 7, paralleling delayed p-tau accumulation. Astrocytic activation on day 1 and AQP4 depolarization between 6 h and day 3 coincided with early cerebral blood flow reduction, biphasic ICP elevation, and transient edema. Together, early vascular and pressure disturbances drive sustained astrocytic and glymphatic dysfunction, defining a critical therapeutic window during days 1–3 post-SAH [58].

The temporal pattern was further investigated in a longer time frame in vivo using contrast-enhanced MRI in a beagle SAH model of endovascular perforation [21]. SAH caused an immediate, severe reduction in glymphatic function, with impaired contrast Gadolinium diethylenetriamine pentaacetic acid (Gd-DTPA) influx and efflux detectable within 1 h, being most pronounced on the ipsilateral side of the brain. Dysfunction persisted for 1 week with partial recovery by 2 weeks, coinciding with acute clot formation, transient arterial injury, early CSF pressure elevation, progressive hydrocephalus, and neurological deficits that peak at day 1 and partially recover by 1–2 weeks, implicating sustained glymphatic collapse in post-SAH hydrocephalus and delayed dysfunction [21].

In a clinical setting, a prospective study of 27 SAH patients used intrathecal contrast gadobutrol and multiphase T1-weighted MRI to examine glymphatic function from <3 months, 3 to 6 months, 6 to 12 months, or >12 months after bleeding [29]. Patients showed markedly reduced glymphatic enrichment, especially after 24 h. The glymphatic impairment was associated with abnormal tracer redistribution from subarachnoid spaces toward the ventricles, indicating disrupted CSF flow. The glymphatic and perivascular transport deficits were most severe at 3–6 months and only partially improved after 12 months, with considerable interpatient variability [29].

##### mLV Pathway

In a mouse SAH model of prechiasmatic cisternal blood injection, lymphatic function was evaluated by tracking fluorescent bead drainage to dcLNs histologically [20]. Bead outflow dropped sharply by 3 h and remained reduced for 72 h, accompanied by structural damage and the diminished drainage capacity of mLVs. Endothelial clustering and flow cytometry revealed a significant loss of meningeal lymphatic endothelial cells, most pronounced at 24 h. These mLV injuries were associated with worse neurological scores, prolonged behavioral deficits, and delayed recovery [20].

In the beagle SAH model, Wang et al. also used contract-enhanced MRI to reveal transient mLVs drainage dysfunction with a time course distinct from glymphatic impairment. The outflow along the olfactory, optic, and cranial nerves was disrupted for about 1 week and largely recovered by 2 weeks. Despite early pathway injury, hemoglobin increased in cervical and sacral lymph nodes at day 1, indicating ongoing lymphatic clearance, and was normalized by 2 weeks [21].

#### 3.2.2. Early Brain Injury (EBI)

EBI occurring within the first 72 h after SAH is increasingly recognized as a critical determinant of neurological outcomes [88,89]. It consists of secondary injuries such as microcirculatory dysfunction, BBB disruption, inflammation, and oxidative cascades that all ultimately lead to cell death [88,90]. Brain lymphatic dysfunction has been implicated as a mechanistic link between the acute insults of SAH and the EBI evolution.

In a mouse SAH model of prechiasmatic cistern blood injection, glymphatic function was first evaluated in vivo using contrast-enhanced MRI after intracisternal injection of a gadolinium chelate [10]. There was a significantly impaired glymphatic flow at 24 h, with contras agent enhancement restricted to the cerebellum and absent in the forebrain, indicating blocked periarterial influx. Fluorescein histology ex vivo confirmed this obstruction [10]. The same group extended these findings to non-human primates, demonstrating that severe glymphatic dysfunction was observed within 20 min of neuronavigation-guided subarachnoid autologous blood injection. An MRI showed markedly reduced parenchymal contrast distribution, with decreased enhanced brain volume and minimal anterior tracer influx, most pronounced ipsilateral to the hemorrhage [12]. Reduced CSF influx and impaired glymphatic tracer clearance promote the accumulation of damage-associated molecular patterns (DAMPs), oxidative stress, and neuroinflammation, contributing to neurological deficit [13,15]. The exacerbation of the glymphatic system following AQP4 knockout worsens EBI in SAH rats [17]. Intracerebroventricular tissue plasminogen activator (tPA), given 15 min post-SAH, partially restored glymphatic flow at 24 h [10] and alleviated neuroinflammation and edema [67] in SAH mice. A calcium channel antagonist, nimodipine, attenuates EBI after SAH in mice in part by preserving glymphatic function [59]. In SAH rats, treatment with either pituitary adenylate cyclase-activating polypeptide (PACAP) [15] or selective caspase-1 inhibitor VX-765 [68] attenuated neurological deficits, likely in part via improving glymphatic dysfunction.

After SAH, erythrocytes rapidly enter perivascular spaces and are cleared by leptomeningeal and perivascular macrophages. Given their close interaction with these cells and role as a major CSF outflow route, mLVs form a key drainage–immune axis for blood clearance and neuroinflammatory regulation [19]. mLV dysfunction exacerbates neuroinflammation via several mechanisms. First, impaired mLV drainage limits the removal of neurotoxic proteins and metabolic byproducts, resulting in their accumulation and prolonged microglial activation [8,91]. Second, defective lymphatic outflow disrupts the normal egress of immune cells such as T cells and macrophages from the meninges, leading to their aberrant retention and accumulation [8,13,92]. Third, the compromised transport of brain-derived antigens to peripheral lymphoid tissues may promote immune dysregulation, leading to an exaggerated systemic inflammatory response [8,21,60]. In SAH models, impaired mLV drainage is associated with immune cell retention, cytokine accumulation, and cerebral edema, whereas enhancing lymphatic outflow improves neuroinflammation and neurological recovery [13]. mLV ablation reduces erythrocyte drainage to dcLNs, prolongs clot persistence [14], worsens cortical perfusion, and increases edema, neuroinflammation, neuronal apoptosis, and neurological deficits [18] in SAH mice. Similarly, animal studies have shown that the blockade of cervical lymphatic drainage exacerbates cerebral blood flow reduction, elevates ICP, and increases edema and oxidative injury, highlighting the role of impaired lymphatic outflow in secondary injury after SAH [62,93].

Meningeal and parenchymal T cell accumulation reflects impaired lymphatic clearance and blood-derived immune entry, linking mLV–dcLN disruption to vasospasm, apoptosis, reduced glymphatic influx, and worse behavioral outcomes after SAH [13]. Spatiotemporal mapping indicates that monocyte-derived macrophages and self-recruiting neutrophils drive acute mLV impairment, while placental growth factor (PGF) promotes early meningeal lymphatic repair after SAH in mice [94]. VEGF-C is essential for meningeal lymphangiogenesis and maintaining the integrity and survival of lymphatic vessels [8,95]. In animal models, VEGF-C pretreatment enhanced meningeal lymphatic drainage and improved neurological outcomes, highlighting lymphatic modulation as a potential therapeutic strategy after SAH [62]. In a mouse SAH model of endovascular perforation, mLV ablation, C-C chemokine receptor type 7 (CCR7) knockout or the intracisternal C–C motif chemokine ligand 21 (CCL21) antibody impaired Th17 clearance, increased meningeal Th17 accumulation, and worsened neuroinflammation and neurological deficits at 24 h. Conversely, VEGF-C or CCL21 protein enhanced lymphatic drainage to dcLN, reduced inflammatory cytokines, and improved neurological outcomes [92].

#### 3.2.3. Delayed Cerebral Ischemia (DCI)

DCI, occurring in 20–30% of SAH patients at 4–14 days after onset, is a major predictor of poor outcomes [96]. In addition to the early focus on the cerebral vasospasm in the large artery, complex multifactorial mechanisms have been proposed, including glymphatic dysfunction and mLV impairment [91,97,98,99,100].

Animal studies have shown that persistent glymphatic dysfunction impairs CSF-ISF exchange, resulting in neurotoxic waste accumulation, sustained inflammation, and vascular disruption, contributing to microthrombi formation, microvasospasm, and intracranial pressure spikes after SAH [11,13,21,23,64,91,98,100]. The sustained microvasospasm triggers spreading cortical depolarizations and may ultimately promote DCI [11,13,91,98]. Glymphatic impairment is greater in the hemisphere ipsilateral to blood injection [10,21] in animal models, consistent with the clinical pattern of DCI in SAH patients [101], suggesting that regional glymphatic dysfunction may contribute to its spatial development and distribution.

In experiment models, intraventricular fibrinolysis restores glymphatic function, improves microvascular flow, and partially normalizes ICP, supporting the concept that the early targeting of glymphatic obstruction may limit DCI severity after SAH [10,11,12].

#### 3.2.4. Cognitive Deficit

SAH survivors often have lasting functional and cognitive deficits that reduce their quality of life and limit their return to work [22,102]. Post-SAH cognitive impairment arises from blood-driven neuroinflammation and cell death, vasospasm/DCI-related ischemia, and microcirculatory injury [22].

Glymphatic dysfunction reduces the clearance of neurotoxic metabolites, contributing to cognitive impairment in neurodegenerative diseases [103], potentially underlying the progressive cognitive decline observed after SAH. In SAH models, glymphatic dysfunction is linked to p-tau accumulation, impaired waste clearance [58], and T cell infiltration, accompanied by hippocampal microvascular spasm, glial activation, neuroinflammation, and neuronal apoptosis [13], forming a feed-forward cycle that amplifies neurodegeneration after SAH. Long-term intraperitoneal β-hydroxybutyrate (BHB) administration alleviated neurological deficits at 28 days in SAH mice by restoring glymphatic function and reducing neuroinflammation, possibly via histone deacetylases’ (HDACs’) inhibition-mediated upregulation of Syntrophin alpha 1 (SNTA1) and restoration of perivascular AQP4 polarity [61].

Age-related mLV dysfunction accelerates amyloid-β accumulation, worsening Alzheimer’s pathology [56]. Similarly, in mouse SAH models, lymphatic ablation exacerbated progressive cognitive impairment and hippocampal neuronal loss, which were evident at 1 month and persisted to 2 months post-SAH [22]. VEGF-C preserved lymphatic integrity via PI3K–AKT signaling, reduced amyloid-β accumulation, and improved cognition, highlighting meningeal lymphatic dysfunction as a therapeutic target after SAH [22]. Dobutamine enhanced CSF outflow and accelerated the mLV-mediated clearance of subarachnoid blood and its breakdown products, reducing neuronal death and improving cognitive outcomes in SAH mice [104]. In a beagle SAH model, intermittent cisterna magna CSF drainage during days 1–3 post-SAH significantly improved neurological and cognitive outcomes, likely by accelerating the recovery of glymphatic and meningeal lymphatic function [21].

#### 3.2.5. Hydrocephalus

Hydrocephalus is a common and devastating complication of SAH, occurring during both the acute and chronic phases and contributing substantially to long-term neurological morbidity and cognitive decline [105]. Persistent glymphatic and mLV dysfunction after SAH promotes CSF stagnation, impairs ISF clearance, worsens neuroinflammation, and increases resistance to ventricular outflow, leading to ventricular enlargement [13,21,106].

In animal models of SAH, intracerebroventricular tissue factor (TF) blockade reduced fibrin and enhanced CSF tracer distribution, implicating TF-mediated coagulation in glymphatic dysfunction and hydrocephalus [66]. Selective caspase-1 inhibitor VX-765 reduced astrocytic TF release, improved CSF clearance, prevented hippocampal loss and hydrocephalus, and attenuated cognitive deficits after SAH in rats [68]. Beyond EBI benefits, daily intermittent cistern magna CSF drainage for 3 days also improved long-term outcomes in a beagle SAH model, reducing ventricular dilation and PVS enlargement by restoring glymphatic and meningeal lymphatic functions [21].

In disease models with impaired CSF dynamics, such as craniosynostosis, mLVs are malformed or dysfunctional, contributing to elevated ICP and reduced CSF flow [107]. Piezo-type mechanosensitive ion channel component 1 (Piezo1) in lymphatic endothelial cells translates mechanical cues from fluid flow into signals that regulate mLVs development and function [107]. Piezo1 agonist Yoda-1 enhances meningeal lymphangiogenesis, CSF drainage to (dcLNs), and brain–CSF exchange, and lowers ICP [8].

In the experimental setting of SAH, however, Piezo1 inhibition shows protective effects [108,109]. Intracranial hypertension induces Piezo1 expression, activates Hippo signaling, and promotes neuronal apoptosis; Piezo1 inhibition improves short- and long-term neurological outcomes in SAH rats [109]. The same group further developed an endoplasmic reticulum membrane-based nanomedicine (CAQKERM@GsMTx4) for targeted Piezo1 inhibition in hemorrhagic regions, which outperformed cell membrane-based vesicles, in vivo and in vitro, suppressed Piezo1 activity, shifted microglia from M1 to M2 phenotypes, and reduced neuroinflammation and neuronal injury [108]. Whether persistent ICP elevation and microvasospasm sustain Piezo1 overactivation and thereby contribute to hydrocephalus formation after SAH requires further investigation.

Disrupting VEGF-C/VEGFR3 signaling impairs mLVs development and CSF drainage to dcLNs [39]. Conversely, intrathecal AAV-VEGF-C pretreatment for 4 weeks enhanced mLV density, CSF drainage, and VEGFR3 signaling, suppressing pro-inflammatory microglia and improving outcomes after ischemic stroke in mice [110]. VEGF-C reduces oxyhemoglobin-induced lymphatic endothelial apoptosis in vitro, limits hippocampal amyloid-β deposition, improves cognition, and exerts mLV protection via PI3K–AKT signaling in SAH mice [22]. These findings suggest that VEGF-C may also represent a potential therapeutic target to prevent or mitigate post-SAH hydrocephalus.

#### 3.2.6. Central-Peripheral Immune Interaction

SAH is significantly associated with non-neurologic medical complications, in which the related mechanisms include the activation of the sympathetic nervous system, the release of catecholamines and other hormones, and inflammatory responses [111]. The glymphatic system provides a functional link between the CNS and the peripheral immune system, enabling coordinated innate and adaptive immune responses during neuroinflammation [9,112]. The drainage of CSF–ISF through mLVs to dcLNs transports immune cells and CNS-derived antigens, promoting peripheral immune activation and potentially contributing to CNS autoimmunity [113]. In injured brains, the activation of effector and regulatory T cells in brain-draining lymph nodes further modulates immune responses within and outside the CNS [56,112,114].

Glymphatic dysfunction after SAH disrupts the brain–lung axis, allowing inflammatory mediators and blood breakdown products into the circulation, damaging the pulmonary endothelium, activating lung immunity, and increasing the risk of acute lung injury and neurogenic pulmonary edema [60]. This highlights SAH as a multisystem disorder and suggests that targeting glymphatic/meningeal lymphatic dysfunction may improve both neurological and respiratory outcomes [60].

### 3.3. Clinical Translation and Limitations

MRI image is most commonly used technique to evaluate glymphatic system and mLVs in human. This includes diffusion tensor image (DTI) analysis along the perivascular index (ALPI) as an indirect measure of perivascular diffusion [34], T2-weight imaging (T2WI) to assess enlarged perivascular spaces (EPVSs) as structural surrogate [42,115] and contrast-enhanced T1-weighted imaging (T1WI) using intrathecal or venous administration gadolinium-based contrast agents [33]. These MRI biomarkers are used to examine not only the interstitial fluid movement in the brain parenchyma, but also the fluid dynamics in the perivascular and subarachnoid spaces, as well as the parasagittal dura and meningeal lymphatics [33].

In patients with SAH, MRI with intrathecal gadolinium showed the significant impairment of glymphatic function throughout the brain 24 h post contrast agent injection, particularly in the cerebral cortex and subcortical white matter, which persisted for 3–6 months and remitted after 12 months [29]. The intrathecal contrast-enhanced MRI shows that glymphatic flow remains impaired for up to 30 days after SAH in patients, independent of the bleed size, and is associated with fibrin deposition on the brain surface [29]. EPVSs in the basal ganglia (BG), rather than the centrum semiovale (CSO), are associated with aneurysmal SAH, even in patients without vascular risk factors, implicating skull-base glymphatic dysfunction [116]. BG-EPVS are also linked to unfavorable outcomes, DCI, and subacute hydrocephalus, whereas only CSO-EPVS independently predict 3-month cognitive impairment [117]. The presence of EPVSs in the centrum semiovale and basal ganglia in patients within 1 month of aSAH onset suggests an association with glymphatic dysfunction [118].

In SAH patients, overlapping high-density CT areas suggest ferritin accumulation along vessels, while HE staining shows blood deposition in paravascular spaces, confirming PVS perfusion in humans [11]. Clinically, approaches such as intraventricular tPA administration [119,120,121] and CSF drainage or flushing [122] have been explored to accelerate clot resolution and improve outcomes prognosis. Such interventions may facilitate the restoration of impaired fluid transport and waste clearance after SAH, leading to better short-term and long-term outcomes. Although favorable effects on clot reduction have been reported, head-motion therapy combined with intraventricular fibrinolysis did not improve delayed cerebral ischemia (DCI) or functional outcomes despite demonstrating safety [119]. These findings indicate that further evaluation in larger cohorts with stricter patient selection criteria is warranted.

Emerging clinical evidence from CSF and blood analyses in patients with SAH support the identification of candidate biomarkers associated with inflammation, coagulation, and clinical outcomes. These findings provide a preliminary basis for patient stratification and may inform future investigations into translational therapeutic strategies targeting brain clearance pathways; however, current evidence remains largely associative and requires further validation.

Elevated Th17 cells and pro-inflammatory mediators in CSF and blood indicate immune-driven neuroinflammation as a modifiable target in post-SAH mLV dysfunction [102]. Preclinical studies show VEGF-C or CCL21 enhances lymphatic drainage to dcLNs, reduces meningeal Th17 accumulation, and improves outcomes, supporting their potential as therapeutic targets and biomarkers for stratification and treatment monitoring [92]. In parallel, early increases in CSF cytokines and coagulation-related factors have been shown to predict the development of chronic hydrocephalus, supporting a role for inflammation–coagulation coupling in impaired CSF circulation and clearance [67].

Neuroinflammation and coagulation pathways are activated in the cerebrospinal fluid (CSF) of patients after SAH. Caspase-1 levels have been reported to correlate with components of the extrinsic coagulation pathway [68]. In preclinical SAH models, the selective caspase-1 inhibitor VX-765 demonstrated beneficial effects through improving glymphatic dysfunction [68]. VX-765 has undergone clinical evaluation and was found to be safe and well-tolerated in Phase I studies, as well as in Phase II trials for epilepsy (NCT01501383), supporting its potential for further investigation in SAH patients.

THBS1 levels are elevated in the CSF of SAH patients and are associated with clinical outcomes, supporting its potential as a prognostic biomarker [20]. Targeting its interaction with the receptor CD47 has shown protective effects on meningeal lymphatic endothelial survival in SAH mice, supporting further investigation for this clinical translation. S100A6, which disrupts lymphatic endothelial tight junctions in cancer models, is also increased in CSF after SAH and correlates with poor outcomes, suggesting it as a candidate biomarker [20].

Overall, these findings support the concept that the modulation of inflammation, coagulation, and CSF circulation may offer clinically relevant avenues to influence brain clearance systems after SAH. However, current evidence remains largely associative, and further studies are required to determine whether these strategies can directly restore glymphatic and meningeal lymphatic function and translate into improved neurological outcomes.

Translating the findings of glymphatic and mLV dysfunction from animal models of SAH into clinical practice is conceptually compelling. However, most preclinical studies rely on small animals (primarily rodents), which exhibit higher mass-specific metabolic rates and accelerated physiological processes compared with humans. Consequently, the timeline of pathological events after stroke, such as inflammation, tissue injury, and cellular senescence, is markedly compressed, with changes occurring over days or weeks in animals potentially corresponding to much later stages in human patients [123]. This discrepancy may partly explain why MRI-based assessments in patients often detect glymphatic alterations at relatively later timepoints following SAH.

In addition to metabolic differences, animal studies typically employ tightly controlled experimental designs with precise timepoint sampling, enabling the clear characterization of early glymphatic and mLV dysfunction. In contrast, clinical imaging is intermittent and frequently delayed, with the hyperacute phase (<6–12 h) rarely captured. As a result, early pathological changes and potential therapeutic windows may be missed in patients. Therefore, the mismatch in temporal resolution between preclinical models and clinical practice should be carefully considered when interpreting and translating findings into human studies.

## 4. Future Directions

The growing recognition of glymphatic and mLV dysfunction as important contributors to SAH pathophysiology opens multiple avenues for future investigation, spanning mechanistic discovery, biomarker development, and therapeutic translation. Addressing these gaps will be essential to move beyond vasospasm-centric paradigms and toward disease-modifying strategies that improve both acute and long-term outcomes in the setting of SAH.

Future basic science research in clinically relevant models may help define cell-specific contributions from astrocytes, endothelial cells, pericytes, and the venous and lymphatic endothelium. Lineage reporters and single-cell/spatial omics will help identify spatial and temporal patterns in contributing to glymphatic and mLV dysfunction and immune regulation.

Emerging clinical and experimental data highlight venous congestion and perivenous efflux failure as contributors to post-SAH clearance dysfunction, challenging an arterial-centric view. Integrating venous hemodynamics with lymphatic outflow using advanced imaging (e.g., MR venography and dynamic CSF tracer studies) may clarify how venous congestion and lymphatic overload jointly regulate clearance efficiency and DCI risk. Translation will require robust, noninvasive biomarkers of CSF–ISF exchange, venous outflow resistance, and meningeal lymphatic drainage, alongside CSF/blood markers of astrocytic polarity, endothelial injury, inflammation, and lymphatic dysfunction for early risk stratification and trial endpoints.

Mechanosensitive pathways, particularly Piezo1, link intracranial pressure surges, vascular stretch, and perivascular edema to sustained clearance failure and hydrocephalus; defining how Piezo1 signaling regulates AQP4 polarity, endothelial integrity, and venous compliance may reveal new intervention points.

## 5. Conclusions

Glymphatic system and the mLV pathway play critical roles in SAH pathophysiology by facilitating CSF circulation, clearing extravasated blood and neurotoxic metabolites, regulating antigen trafficking, and shaping neuroimmune signaling. The persistent failure of glymphatic–lymphatic clearance provides a unifying mechanism linking early brain injury to delayed cerebral ischemia, hydrocephalus, and long-term cognitive impairment, consistent with contemporary views that delayed injury is multifactorial and microvascular- and immune-driven. Translational priorities include validated imaging and biomarkers of clearance dysfunction, and therapeutic strategies that restore glymphatic and mLV function, shifting SAH care from complication management toward disease modification.

## Data Availability

No new data were created or analyzed in this study. Data sharing is not applicable to this article.

## References

[1] Sanicola H.W., Stewart C.E., Luther P., Yabut K., Guthikonda B., Jordan J.D., Alexander J.S. (2023). Pathophysiology, Management, and Therapeutics in Subarachnoid Hemorrhage and Delayed Cerebral Ischemia: An Overview. Pathophysiology.

[2] Iliff J.J., Wang M., Liao Y., Plogg B.A., Peng W., Gundersen G.A., Benveniste H., Vates G.E., Deane R., Goldman S.A. (2012). A paravascular pathway facilitates CSF flow through the brain parenchyma and the clearance of interstitial solutes, including amyloid beta. Sci. Transl. Med..

[3] Iliff J.J., Wang M., Zeppenfeld D.M., Venkataraman A., Plog B.A., Liao Y., Deane R., Nedergaard M. (2013). Cerebral arterial pulsation drives paravascular CSF-interstitial fluid exchange in the murine brain. J. Neurosci..

[4] Mestre H., Tithof J., Du T., Song W., Peng W., Sweeney A.M., Olveda G., Thomas J.H., Nedergaard M., Kelley D.H. (2018). Flow of cerebrospinal fluid is driven by arterial pulsations and is reduced in hypertension. Nat. Commun..

[5] Aspelund A., Antila S., Proulx S.T., Karlsen T.V., Karaman S., Detmar M., Wiig H., Alitalo K. (2015). A dural lymphatic vascular system that drains brain interstitial fluid and macromolecules. J. Exp. Med..

[6] Louveau A., Smirnov I., Keyes T.J., Eccles J.D., Rouhani S.J., Peske J.D., Derecki N.C., Castle D., Mandell J.W., Lee K.S. (2015). Structural and functional features of central nervous system lymphatic vessels. Nature.

[7] Salvador A.F.M., Abduljawad N., Kipnis J. (2024). Meningeal Lymphatics in Central Nervous System Diseases. Annu. Rev. Neurosci..

[8] Zhang Q., Niu Y., Li Y., Xia C., Chen Z., Chen Y., Feng H. (2025). Meningeal lymphatic drainage: Novel insights into central nervous system disease. Signal Transduct. Target. Ther..

[9] Hablitz L.M., Nedergaard M. (2021). The Glymphatic System: A Novel Component of Fundamental Neurobiology. J. Neurosci..

[10] Gaberel T., Gakuba C., Goulay R., Martinez De Lizarrondo S., Hanouz J.L., Emery E., Touze E., Vivien D., Gauberti M. (2014). Impaired glymphatic perfusion after strokes revealed by contrast-enhanced MRI: A new target for fibrinolysis?. Stroke.

[11] Luo C., Yao X., Li J., He B., Liu Q., Ren H., Liang F., Li M., Lin H., Peng J. (2016). Paravascular pathways contribute to vasculitis and neuroinflammation after subarachnoid hemorrhage independently of glymphatic control. Cell Death Dis..

[12] Goulay R., Flament J., Gauberti M., Naveau M., Pasquet N., Gakuba C., Emery E., Hantraye P., Vivien D., Aron-Badin R. (2017). Subarachnoid Hemorrhage Severely Impairs Brain Parenchymal Cerebrospinal Fluid Circulation in Nonhuman Primate. Stroke.

[13] Pu T., Zou W., Feng W., Zhang Y., Wang L., Wang H., Xiao M. (2019). Persistent Malfunction of Glymphatic and Meningeal Lymphatic Drainage in a Mouse Model of Subarachnoid Hemorrhage. Exp. Neurobiol..

[14] Chen J., Wang L., Xu H., Xing L., Zhuang Z., Zheng Y., Li X., Wang C., Chen S., Guo Z. (2020). Meningeal lymphatics clear erythrocytes that arise from subarachnoid hemorrhage. Nat. Commun..

[15] Fang Y., Shi H., Ren R., Huang L., Okada T., Lenahan C., Gamdzyk M., Travis Z.D., Lu Q., Tang L. (2020). Pituitary Adenylate Cyclase-Activating Polypeptide Attenuates Brain Edema by Protecting Blood-Brain Barrier and Glymphatic System After Subarachnoid Hemorrhage in Rats. Neurotherapeutics.

[16] Liu E., Peng X., Ma H., Zhang Y., Yang X., Zhang Y., Sun L., Yan J. (2020). The Involvement of Aquaporin-4 in the Interstitial Fluid Drainage Impairment Following Subarachnoid Hemorrhage. Front. Aging Neurosci..

[17] Liu E., Sun L., Zhang Y., Wang A., Yan J. (2020). Aquaporin4 Knockout Aggravates Early Brain Injury Following Subarachnoid Hemorrhage Through Impairment of the Glymphatic System in Rat Brain. Acta Neurochir. Suppl..

[18] Liu Q., Hou C., Zhang H., Fu C., Wang W., Wang B., Li J., Zhao Y., Yang X. (2021). Impaired meningeal lymphatic vessels exacerbate early brain injury after experimental subarachnoid hemorrhage. Brain Res..

[19] Wan H., Brathwaite S., Ai J., Hynynen K., Macdonald R.L. (2021). Role of perivascular and meningeal macrophages in outcome following experimental subarachnoid hemorrhage. J. Cereb. Blood Flow Metab..

[20] Wang X., Zhang A., Yu Q., Wang Z., Wang J., Xu P., Liu Y., Lu J., Zheng J., Li H. (2023). Single-Cell RNA Sequencing and Spatial Transcriptomics Reveal Pathogenesis of Meningeal Lymphatic Dysfunction after Experimental Subarachnoid Hemorrhage. Adv. Sci..

[21] Wang J., Lv T., Jia F., Li Y., Ma W., Xiao Z.P., Yu W., Zhao H., Zhang X., Hu Q. (2024). Subarachnoid hemorrhage distinctively disrupts the glymphatic and meningeal lymphatic systems in beagles. Theranostics.

[22] Cai Y., Shao Y., Yuan H., Feng L., Wang J., Yang M., Li C., Sun B., Mao L. (2025). Meningeal lymphatic dysfunction drives cognitive impairment after experimental subarachnoid hemorrhage. Neurotherapeutics.

[23] Mestre H., Kostrikov S., Mehta R.I., Nedergaard M. (2017). Perivascular spaces, glymphatic dysfunction, and small vessel disease. Clin. Sci..

[24] Rasmussen M.K., Mestre H., Nedergaard M. (2018). The glymphatic pathway in neurological disorders. Lancet Neurol..

[25] Zeppenfeld D.M., Simon M., Haswell J.D., D’Abreo D., Murchison C., Quinn J.F., Grafe M.R., Woltjer R.L., Kaye J., Iliff J.J. (2017). Association of Perivascular Localization of Aquaporin-4 With Cognition and Alzheimer Disease in Aging Brains. JAMA Neurol..

[26] Absinta M., Ha S.K., Nair G., Sati P., Luciano N.J., Palisoc M., Louveau A., Zaghloul K.A., Pittaluga S., Kipnis J. (2017). Human and nonhuman primate meninges harbor lymphatic vessels that can be visualized noninvasively by MRI. Elife.

[27] Eide P.K., Hovd M., Valnes L.M., Pripp A., Ringstad G. (2026). When blood hits the brain: Altered glymphatic and dural lymphatic function after surface bleeds. Acta Neurochir..

[28] Eide P.K., Ringstad G. (2015). MRI with intrathecal MRI gadolinium contrast medium administration: A possible method to assess glymphatic function in human brain. Acta Radiol. Open.

[29] Eide P.K., Undseth R.M., Pripp A., Lashkarivand A., Nedregaard B., Sletteberg R., Ronning P.A., Sorteberg A.G., Ringstad G., Valnes L.M. (2025). Impact of Subarachnoid Hemorrhage on Human Glymphatic Function: A Time-Evolution Magnetic Resonance Imaging Study. Stroke.

[30] Kamagata K., Andica C., Takabayashi K., Saito Y., Taoka T., Nozaki H., Kikuta J., Fujita S., Hagiwara A., Kamiya K. (2022). Association of MRI Indices of Glymphatic System With Amyloid Deposition and Cognition in Mild Cognitive Impairment and Alzheimer Disease. Neurology.

[31] Kiviniemi V., Wang X., Korhonen V., Keinanen T., Tuovinen T., Autio J., LeVan P., Keilholz S., Zang Y.F., Hennig J. (2016). Ultra-fast magnetic resonance encephalography of physiological brain activity—Glymphatic pulsation mechanisms?. J. Cereb. Blood Flow Metab..

[32] Naganawa S., Nakane T., Kawai H., Taoka T. (2017). Gd-based Contrast Enhancement of the Perivascular Spaces in the Basal Ganglia. Magn. Reson. Med. Sci..

[33] Naganawa S., Taoka T., Ito R., Kawamura M. (2024). The Glymphatic System in Humans: Investigations With Magnetic Resonance Imaging. Investig. Radiol..

[34] Taoka T., Masutani Y., Kawai H., Nakane T., Matsuoka K., Yasuno F., Kishimoto T., Naganawa S. (2017). Evaluation of glymphatic system activity with the diffusion MR technique: Diffusion tensor image analysis along the perivascular space (DTI-ALPS) in Alzheimer’s disease cases. Jpn. J. Radiol..

[35] Yamamoto E.A., Bagley J.H., Geltzeiler M., Sanusi O.R., Dogan A., Liu J.J., Piantino J. (2024). The perivascular space is a conduit for cerebrospinal fluid flow in humans: A proof-of-principle report. Proc. Natl. Acad. Sci. USA.

[36] Simon M., Wang M.X., Ismail O., Braun M., Schindler A.G., Reemmer J., Wang Z., Haveliwala M.A., O’Boyle R.P., Han W.Y. (2022). Loss of perivascular aquaporin-4 localization impairs glymphatic exchange and promotes amyloid beta plaque formation in mice. Alzheimers Res. Ther..

[37] Ding Z., Fan X., Zhang Y., Yao M., Wang G., Dong Y., Liu J., Song W. (2023). The glymphatic system: A new perspective on brain diseases. Front. Aging Neurosci..

[38] Mestre H., Mori Y., Nedergaard M. (2020). The Brain’s Glymphatic System: Current Controversies. Trends Neurosci..

[39] Antila S., Karaman S., Nurmi H., Airavaara M., Voutilainen M.H., Mathivet T., Chilov D., Li Z., Koppinen T., Park J.H. (2017). Development and plasticity of meningeal lymphatic vessels. J. Exp. Med..

[40] Izen R.M., Yamazaki T., Nishinaka-Arai Y., Hong Y.K., Mukouyama Y.S. (2018). Postnatal development of lymphatic vasculature in the brain meninges. Dev. Dyn..

[41] Shulyatnikova T., Hayden M.R. (2023). Why Are Perivascular Spaces Important?. Medicina.

[42] Wardlaw J.M., Benveniste H., Nedergaard M., Zlokovic B.V., Mestre H., Lee H., Doubal F.N., Brown R., Ramirez J., MacIntosh B.J. (2020). Perivascular spaces in the brain: Anatomy, physiology and pathology. Nat. Rev. Neurol..

[43] Lv T., Zhao B., Hu Q., Zhang X. (2021). The Glymphatic System: A Novel Therapeutic Target for Stroke Treatment. Front. Aging Neurosci..

[44] Zheng Z., Chopp M., Chen J. (2020). Multifaceted roles of pericytes in central nervous system homeostasis and disease. J. Cereb. Blood Flow Metab..

[45] Dabija M.G., Tataru C.I., Dumitru A.V., Munteanu O., Radoi M.P., Ciurea A.V., Petrescu I.A. (2025). Noradrenergic Slow Vasomotion: The Hidden Fluid Pump Linking Sleep, Brain Clearance, and Dementia Pathogenesis. Int. J. Mol. Sci..

[46] Xie L., Kang H., Xu Q., Chen M.J., Liao Y., Thiyagarajan M., O’Donnell J., Christensen D.J., Nicholson C., Iliff J.J. (2013). Sleep drives metabolite clearance from the adult brain. Science.

[47] Muccio M., Sun Z., Chu D., Damadian B.E., Minkoff L., Bonanni L., Ge Y. (2024). The impact of body position on neurofluid dynamics: Present insights and advancements in imaging. Front. Aging Neurosci..

[48] Strbacko I., Rados M., Jurjevic I., Oreskovic D., Klarica M. (2024). Body position influence on cerebrospinal fluid volume redistribution inside the cranial and spinal CSF compartments. Front. Hum. Neurosci..

[49] Vera Quesada C.L., Rao S.B., Torp R., Eide P.K. (2023). Widespread distribution of lymphatic vessels in human dura mater remote from sinus veins. Front. Cell Dev. Biol..

[50] Giff A.E., Clark M.W., Bhattacharyya S., Sage P.T., Madore B., Guenette J.P., Miyawaki E.K. (2026). Deep cervical lymph node analysis in central nervous system inflammatory disease. Front. Immunol..

[51] Jacob L., de Brito Neto J., Lenck S., Corcy C., Benbelkacem F., Geraldo L.H., Xu Y., Thomas J.M., El Kamouh M.R., Spajer M. (2022). Conserved meningeal lymphatic drainage circuits in mice and humans. J. Exp. Med..

[52] Moazen M., Alazmani A., Rafferty K., Liu Z.J., Gustafson J., Cunningham M.L., Fagan M.J., Herring S.W. (2016). Intracranial pressure changes during mouse development. J. Biomech..

[53] Ahn J.H., Cho H., Kim J.H., Kim S.H., Ham J.S., Park I., Suh S.H., Hong S.P., Song J.H., Hong Y.K. (2019). Meningeal lymphatic vessels at the skull base drain cerebrospinal fluid. Nature.

[54] Cavdar S., Kose B., Altinoz D., Soyler G., Cingoz A., Gurses I.A., Ozkan M., Asliyuksek H., Cakir H. (2023). Lymphatic Vessels Accompanying Dorsal and Basal Dural Sinuses in the Human Brain. J. Chem. Neuroanat..

[55] Zhao M.Y., Ye C.Y., Liu Y.C., Wang X.M., Fu J.C., Liu X.Y., Zhu R., Li Y.Z., Tian Q. (2025). Role of meningeal lymphatic vessels in brain homeostasis. Front. Immunol..

[56] Da Mesquita S., Fu Z., Kipnis J. (2018). The Meningeal Lymphatic System: A New Player in Neurophysiology. Neuron.

[57] Zhang Z., Fang Q., Zhang Y., Zhu Y., Zhang W., Zhu Y., Deng X. (2022). Magnetic resonance analysis of deep cerebral venous vasospasm after subarachnoid hemorrhage in rabbits. Front. Cardiovasc. Med..

[58] Hou C., Li J., Wang B., Liu Q., Zhao Y., Zhang H., Wang W., Ren W., Cui X., Yang X. (2022). Dynamic Evolution of the Glymphatic System at the Early Stages of Subarachnoid Hemorrhage. Front. Neurol..

[59] Hou C., Liu Q., Zhang H., Wang W., Wang B., Cui X., Li J., Ren W., Yang X. (2022). Nimodipine Attenuates Early Brain Injury by Protecting the Glymphatic System After Subarachnoid Hemorrhage in Mice. Neurochem. Res..

[60] Lee E.C., Oh J.S. (2025). Glymphatic Dysfunction in Neuro-Pulmonary Complications Following Subarachnoid Hemorrhage: A New Perspective on Brain-Lung Axis Disruption. Cells.

[61] Tan X., Li X., Li R., Meng W., Xie Z., Li J., Pang Y., Huang G., Li L., Li H. (2024). beta-hydroxybutyrate alleviates neurological deficits by restoring glymphatic and inflammation after subarachnoid hemorrhage in mice. Exp. Neurol..

[62] Luo S.Q., Gao S.Q., Fei M.X., Xue W., Yan S., Ran Z., Han Y.L., Wang H.D., Zhou M.L. (2024). Ligation of cervical lymphatic vessels decelerates blood clearance and worsens outcomes after experimental subarachnoid hemorrhage. Brain Res..

[63] Siler D.A., Berlow Y.A., Kukino A., Davis C.M., Nelson J.W., Grafe M.R., Ono H., Cetas J.S., Pike M., Alkayed N.J. (2015). Soluble Epoxide Hydrolase in Hydrocephalus, Cerebral Edema, and Vascular Inflammation After Subarachnoid Hemorrhage. Stroke.

[64] Liu J.J., Raskin J.S., McFarlane R., Samatham R., Cetas J.S. (2020). Subarachnoid Hemorrhage Pattern Predicts Acute Cerebral Blood Flow Response in the Rat. Acta Neurochir. Suppl..

[65] Sun Y., Liu E., Pei Y., Yao Q., Ma H., Mu Y., Wang Y., Zhang Y., Yang X., Wang X. (2022). The impairment of intramural periarterial drainage in brain after subarachnoid hemorrhage. Acta Neuropathol. Commun..

[66] Golanov E.V., Bovshik E.I., Wong K.K., Pautler R.G., Foster C.H., Federley R.G., Zhang J.Y., Mancuso J., Wong S.T., Britz G.W. (2018). Subarachnoid hemorrhage—Induced block of cerebrospinal fluid flow: Role of brain coagulation factor III (tissue factor). J. Cereb. Blood Flow Metab..

[67] Fang Y., Liu Y., Chen L., Wang J., Zhang J., Zhang H., Tian S., Zhang A., Zhang J., Zhang J.H. (2024). Cerebrospinal fluid markers of neuroinflammation and coagulation in severe cerebral edema and chronic hydrocephalus after subarachnoid hemorrhage: A prospective study. J. Neuroinflamm..

[68] Fang Y., Wang X., Lu J., Shi H., Huang L., Shao A., Zhang A., Liu Y., Ren R., Lenahan C. (2022). Inhibition of caspase-1-mediated inflammasome activation reduced blood coagulation in cerebrospinal fluid after subarachnoid haemorrhage. EBioMedicine.

[69] Ji Y., Meng Q.H., Wang Z.G. (2014). Changes in the coagulation and fibrinolytic system of patients with subarachnoid hemorrhage. Neurol. Med. Chir. (Tokyo).

[70] Li Y., Wu P., Bihl J.C., Shi H. (2020). Underlying Mechanisms and Potential Therapeutic Molecular Targets in Blood-Brain Barrier Disruption after Subarachnoid Hemorrhage. Curr. Neuropharmacol..

[71] Greene C., Hanley N., Campbell M. (2019). Claudin-5: Gatekeeper of neurological function. Fluids Barriers CNS.

[72] Lenzser G., Szebeni G.J., Balogh F., Gemes N., Schwarcz A., Csecsei P. (2025). Claudin proteins and hemorrhage severity in aneurysmal subarachnoid hemorrhage: Correlation with modified Fisher score but not functional outcome. Neurosurg. Rev..

[73] Verheggen I.C.M., Van Boxtel M.P.J., Verhey F.R.J., Jansen J.F.A., Backes W.H. (2018). Interaction between blood-brain barrier and glymphatic system in solute clearance. Neurosci. Biobehav. Rev..

[74] Li R., Zhao M., Yao D., Zhou X., Lenahan C., Wang L., Ou Y., He Y. (2022). The role of the astrocyte in subarachnoid hemorrhage and its therapeutic implications. Front. Immunol..

[75] Pappas A.C., Koide M., Wellman G.C. (2015). Astrocyte Ca^2+^ Signaling Drives Inversion of Neurovascular Coupling after Subarachnoid Hemorrhage. J. Neurosci..

[76] Koide M., Bonev A.D., Nelson M.T., Wellman G.C. (2012). Inversion of neurovascular coupling by subarachnoid blood depends on large-conductance Ca^2+^-activated K^+^ (BK) channels. Proc. Natl. Acad. Sci. USA.

[77] Wu L., Liu Y., He Q., Ao G., Xu N., He W., Liu X., Huang L., Yu Q., Kanamaru H. (2024). PEDF-34 attenuates neurological deficit and suppresses astrocyte-dependent neuroinflammation by modulating astrocyte polarization via 67LR/JNK/STAT1 signaling pathway after subarachnoid hemorrhage in rats. J. Neuroinflamm..

[78] Addis A., Baggiani M., Citerio G. (2023). Intracranial Pressure Monitoring and Management in Aneurysmal Subarachnoid Hemorrhage. Neurocrit. Care.

[79] Xiang T., Feng D., Zhang X., Chen Y., Wang H., Liu X., Gong Z., Yuan J., Liu M., Sha Z. (2022). Effects of increased intracranial pressure on cerebrospinal fluid influx, cerebral vascular hemodynamic indexes, and cerebrospinal fluid lymphatic efflux. J. Cereb. Blood Flow Metab..

[80] Chen S., Chen Y., Xu L., Matei N., Tang J., Feng H., Zhang J. (2015). Venous system in acute brain injury: Mechanisms of pathophysiological change and function. Exp. Neurol..

[81] Li Q., Khatibi N., Zhang J.H. (2014). Vascular neural network: The importance of vein drainage in stroke. Transl. Stroke Res..

[82] Duman E., Coven I., Yildirim E., Yilmaz C., Pinar H.U., Ozdemir O. (2017). Association Between Brain Venous Drainage, Cerebral Aneurysm Formation and Aneurysm Rupture. Turk. Neurosurg..

[83] Tsai F.Y., Yen A., Guo W.Y., Wu C.C. (2011). Venous hypertension and cerebral aneurysm rupture. Neuroradiol. J..

[84] Mursch K., Wachter A., Radke K., Buhre W., Al-Sufi S., Munzel U., Behnke-Mursch J., Kolenda H. (2001). Blood flow velocities in the basal vein after subarachnoid haemorrhage. A prospective study using transcranial duplex sonography. Acta Neurochir..

[85] Niesen W.D., Rosenkranz M., Schummer W., Weiller C., Sliwka U. (2004). Cerebral venous flow velocity predicts poor outcome in subarachnoid hemorrhage. Stroke.

[86] Lyman K.A., Adusumilli G., Kamdar H.A., Yang J., Rordorf G.A., Rubin D.B., Regenhardt R.W., Stapleton C.J., Patel A.B., Locascio J.J. (2025). Impaired Sphenoparietal Venous Drainage Is Associated With Increased Risk of Vasospasm in Aneurysmal Subarachnoid Hemorrhage. Stroke Vasc. Interv. Neurol..

[87] El Kamouh M.R., Spajer M., Singhabahu R., Sailor K., Bourrienne M.C., Mouton L., Koundal S., Doukhi D., Grine A., Ninnemann J. (2024). Cerebral Venous Blood Flow Regulates Brain Fluid Clearance via Meningeal Lymphatics. bioRxiv.

[88] Fujii M., Yan J., Rolland W.B., Soejima Y., Caner B., Zhang J.H. (2013). Early brain injury, an evolving frontier in subarachnoid hemorrhage research. Transl. Stroke Res..

[89] Tuzi S., Kranawetter B., Mielke D., Rohde V., Malinova V. (2024). Systematic assessment of early brain injury severity at admission with aneurysmal subarachnoid hemorrhage. Neurosurg. Rev..

[90] Lauzier D.C., Jayaraman K., Yuan J.Y., Diwan D., Vellimana A.K., Osbun J.W., Chatterjee A.R., Athiraman U., Dhar R., Zipfel G.J. (2023). Early Brain Injury After Subarachnoid Hemorrhage: Incidence and Mechanisms. Stroke.

[91] Suzuki H., Kanamaru H., Kawakita F., Asada R., Fujimoto M., Shiba M. (2021). Cerebrovascular pathophysiology of delayed cerebral ischemia after aneurysmal subarachnoid hemorrhage. Histol. Histopathol..

[92] Gao D., Zou B., Zhu K., Bi S., Zhang W., Yang X., Lai J., Liang G., Pan P. (2024). Enhancing Th17 cells drainage through meningeal lymphatic vessels alleviate neuroinflammation after subarachnoid hemorrhage. J. Neuroinflamm..

[93] Sun B.L., Xie F.M., Yang M.F., Cao M.Z., Yuan H., Wang H.T., Wang J.R., Jia L. (2011). Blocking cerebral lymphatic drainage deteriorates cerebral oxidative injury in rats with subarachnoid hemorrhage. Acta Neurochir. Suppl..

[94] Zhu B., Liu C., Luo M., Chen J., Tian S., Zhan T., Liu Y., Zhang H., Wang Z., Zhang J. (2025). Spatiotemporal dynamic changes of meningeal microenvironment influence meningeal lymphatic function following subarachnoid hemorrhage: From inflammatory response to tissue remodeling. J. Neuroinflamm..

[95] Da Mesquita S., Louveau A., Vaccari A., Smirnov I., Cornelison R.C., Kingsmore K.M., Contarino C., Onengut-Gumuscu S., Farber E., Raper D. (2018). Functional aspects of meningeal lymphatics in ageing and Alzheimer’s disease. Nature.

[96] McBride D.W., Blackburn S.L., Peeyush K.T., Matsumura K., Zhang J.H. (2017). The Role of Thromboinflammation in Delayed Cerebral Ischemia after Subarachnoid Hemorrhage. Front. Neurol..

[97] Dodd W.S., Laurent D., Dumont A.S., Hasan D.M., Jabbour P.M., Starke R.M., Hosaka K., Polifka A.J., Hoh B.L., Chalouhi N. (2021). Pathophysiology of Delayed Cerebral Ischemia After Subarachnoid Hemorrhage: A Review. J. Am. Heart Assoc..

[98] Motwani K., Dodd W.S., Laurent D., Lucke-Wold B., Chalouhi N. (2022). Delayed cerebral ischemia: A look at the role of endothelial dysfunction, emerging endovascular management, and glymphatic clearance. Clin. Neurol. Neurosurg..

[99] Stragier H., Vandersmissen H., Ordies S., Thiessen S., Mesotten D., Peuskens D., Ten Cate H. (2025). Pathophysiological mechanisms underlying early brain injury and delayed cerebral ischemia in the aftermath of aneurysmal subarachnoid hemorrhage: A comprehensive analysis. Front. Neurol..

[100] Zhou J., Guo P., Guo Z., Sun X., Chen Y., Feng H. (2022). Fluid metabolic pathways after subarachnoid hemorrhage. J. Neurochem..

[101] Hurth H., Steiner J., Birkenhauer U., Roder C., Hauser T.K., Ernemann U., Tatagiba M., Ebner F.H. (2021). Relationship of the vascular territory affected by delayed cerebral ischemia and the location of the ruptured aneurysm in patients with aneurysmal subarachnoid hemorrhage. Neurosurg. Rev..

[102] Geraghty J.R., Lara-Angulo M.N., Spegar M., Reeh J., Testai F.D. (2020). Severe cognitive impairment in aneurysmal subarachnoid hemorrhage: Predictors and relationship to functional outcome. J. Stroke Cerebrovasc. Dis..

[103] Nedergaard M., Goldman S.A. (2020). Glymphatic failure as a final common pathway to dementia. Science.

[104] Wang X., Deng H.J., Gao S.Q., Li T., Gao C.C., Han Y.L., Zhuang Y.S., Qiu J.Y., Miao S.H., Zhou M.L. (2023). Dobutamine promotes the clearance of erythrocytes from the brain to cervical lymph nodes after subarachnoid hemorrhage in mice. Front. Pharmacol..

[105] Kuo L.T., Huang A.P. (2021). The Pathogenesis of Hydrocephalus Following Aneurysmal Subarachnoid Hemorrhage. Int. J. Mol. Sci..

[106] Treffy R.W., Eraky A.M., Hussain O., Hedayat H.S. (2023). Glymphatics for the Neurosurgeon. Neurosurg. Pract..

[107] Matrongolo M.J., Ang P.S., Wu J., Jain A., Thackray J.K., Reddy A., Sung C.C., Barbet G., Hong Y.K., Tischfield M.A. (2023). Piezo1 agonist restores meningeal lymphatic vessels, drainage, and brain-CSF perfusion in craniosynostosis and aged mice. J. Clin. Investig..

[108] Zhang X., Jiang E., Fu W., Wang Y., Wang Y., Fang Z., Zhang Z., Duan J., Zeng J., Yan Y. (2025). Engineered endoplasmic reticulum-targeting nanodrugs with Piezo1 inhibition and promotion of cell uptake for subarachnoid hemorrhage inflammation repair. J. Nanobiotechnol..

[109] Zeng J., Fang Z., Duan J., Zhang Z., Wang Y., Wang Y., Chen L., Wang J., Liu F. (2024). Activation of Piezo1 by intracranial hypertension induced neuronal apoptosis via activating hippo pathway. CNS Neurosci. Ther..

[110] Boisserand L.S.B., Geraldo L.H., Bouchart J., El Kamouh M.R., Lee S., Sanganahalli B.G., Spajer M., Zhang S., Lee S., Parent M. (2024). VEGF-C prophylaxis favors lymphatic drainage and modulates neuroinflammation in a stroke model. J. Exp. Med..

[111] Chen S., Li Q., Wu H., Krafft P.R., Wang Z., Zhang J.H. (2014). The harmful effects of subarachnoid hemorrhage on extracerebral organs. BioMed Res. Int..

[112] Mogensen F.L., Delle C., Nedergaard M. (2021). The Glymphatic System (En)during Inflammation. Int. J. Mol. Sci..

[113] Louveau A., Herz J., Alme M.N., Salvador A.F., Dong M.Q., Viar K.E., Herod S.G., Knopp J., Setliff J.C., Lupi A.L. (2018). CNS lymphatic drainage and neuroinflammation are regulated by meningeal lymphatic vasculature. Nat. Neurosci..

[114] Dikiy S., Rudensky A.Y. (2023). Principles of regulatory T cell function. Immunity.

[115] Ramirez J., Berezuk C., McNeely A.A., Gao F., McLaurin J., Black S.E. (2016). Imaging the Perivascular Space as a Potential Biomarker of Neurovascular and Neurodegenerative Diseases. Cell. Mol. Neurobiol..

[116] Yu Q., Wang H., Zhang W., Zhang X., Zhao J., Gong L., Liu X. (2024). MRI-visible enlarged perivascular spaces in basal ganglia rather than centrum semiovale was associated with aneurysmal subarachnoid hemorrhage. Front. Neurol..

[117] Wang H., Yu Q., Zhang W., Yao S., Zhang Y., Dong Q., Zhao Y., Lin J., Liu X., Gong L. (2025). Enlarged Perivascular Spaces (EPVS) Associated with Functional and Cognitive Outcome After Aneurysm Subarachnoid Hemorrhage (aSAH). Transl. Stroke Res..

[118] Kim J., Joo B., Kim J.W., Park M., Ahn S.J., Park S.K., Suh S.H. (2022). Aggravation of Enlarged Perivascular Spaces in the Centrum Semiovale of Patients with Aneurysmal Subarachnoid Hemorrhage. Clin. Neuroradiol..

[119] Etminan N., Beseoglu K., Eicker S.O., Turowski B., Steiger H.J., Hanggi D. (2013). Prospective, randomized, open-label phase II trial on concomitant intraventricular fibrinolysis and low-frequency rotation after severe subarachnoid hemorrhage. Stroke.

[120] Kramer A.H., Roberts D.J., Holodinsky J., Todd S., Hill M.D., Zygun D.A., Faris P., Wong J.H. (2014). Intraventricular tissue plasminogen activator in subarachnoid hemorrhage patients: A prospective, randomized, placebo-controlled pilot trial. Neurocrit. Care.

[121] Liu C., Wroe W.W., Zeineddine H.A., Dawes B., Giordano M., McCabe A., Chen C.J., McBride D.W., Gusdon A.M., Choi H.A. (2025). Imaging efficacy and safety of low dose intraventricular tissue plasminogen activator in aneurysmal subarachnoid hemorrhage: Case series. Acta Neurochir..

[122] Dufwenberg M.A., Garfinkel A.R., Greenhill M., Garewal A., Larson M.C. (2023). Cerebrospinal fluid flushing as a means of neuroprotection. Front. Neurosci..

[123] Agoston D.V. (2017). How to Translate Time? The Temporal Aspect of Human and Rodent Biology. Front. Neurol..

